# Effects of the Long-Term Administration of Uridine on the Functioning of Rat Liver Mitochondria in Hyperthyroidism

**DOI:** 10.3390/ijms242316730

**Published:** 2023-11-24

**Authors:** Natalya Venediktova, Ilya Solomadin, Anna Nikiforova, Konstantin N. Belosludtsev, Galina Mironova

**Affiliations:** 1 Laboratory of Mitochondrial Transport, Institute of Theoretical and Experimental Biophysics, Russian Academy of Sciences, 142290 Pushchino, Russia; 2 Department of Biochemistry, Cell Biology and Microbiology, Mari State University, pl. Lenina 1, 424001 Yoshkar-Ola, Russia

**Keywords:** mitochondria, energy metabolism, thyroid hormones, mitochondrial dysfunction, uridine

## Abstract

The effect of uridine (30 mg/kg for 7 days; intraperitoneally) on the functions of liver mitochondria in rats with experimentally induced hyperthyroidism (HT) (200 µg/100 g for 7 days, intraperitoneally) is studied in this paper. An excess of thyroid hormones (THs) led to an intensification of energy metabolism, the development of oxidative stress, a significant increase in the biogenesis, and changes in the content of proteins responsible for the fusion and fission of mitochondria. The injection of uridine did not change the concentration of THs in the blood of hyperthyroid rats (HRs) but normalized their body weight. The exposure to uridine improved the parameters of oxidative phosphorylation and corrected the activity of some complexes of the electron transport chain (ETC) in the liver mitochondria of HRs. The analysis of ETC complexes showed that the level of CI–CV did not change by the action of uridine in rats with the condition of HT. The application of uridine caused a significant increase in the activity of superoxide dismutase and lowered the rate of hydrogen peroxide production. It was found that uridine affected mitochondrial biogenesis by increasing the expression of the genes *Ppargc1a* and *NRF1* and diminishing the expression of the *Parkin* gene responsible for mitophagy compared with the control animals. In addition, the mRNA level of the *OPA1* gene was restored, which may indicate an improvement in the ETC activity and oxidative phosphorylation in the mitochondria of HR. As a whole, the results obtained demonstrate that uridine has a protective effect against HT-mediated functional disorders in the metabolism of rat liver mitochondria.

## 1. Introduction

In recent decades, a significant increase has been noted worldwide in the number of patients suffering from diseases associated with the inability of mitochondria to adapt to changing energy demands. This leads to chronic bioenergetic defects and the cumulative destruction of cells, which may promote the development of metabolic and neurogenetic disorders [1]. An example of such pathology is the hyperfunction of the thyroid gland, which is accompanied by the excessive production of the hormones triiodothyronine (T_3_) and thyroxine (T_4_). Normally, THs regulate metabolic processes and the functioning of many organs and systems; they also affect palpitation, respiration, the activity of genital organs, and the gastrointestinal tract. To maintain an adequate amount of ATP synthesis in HT, higher respiration rates are required [2]. As a result, the catabolism of carbohydrates, proteins, and triglycerides in the body is activated, and a hypermetabolic effect develops. This pathological condition can either directly affect the organism or aggravate the course of other diseases: cardiomyopathy, arrhythmia, cardiac insufficiency, diabetes, heat intolerance, insomnia, myasthenia, climacteric, and ageing [3]. In this case, marked functional and structural changes occur in mitochondria. It is known that mitochondrial dysfunction plays an important role in both the development and progression of HT, especially due to the generation of oxidative stress, impaired oxidative phosphorylation, and improper operation of the system of mitochondrial quality control and mitophagy [4,5,6]. 

At present, many laboratories of the world are engaged in intensive studies of the molecular and cellular mechanisms of diseases associated with mitochondrial dysfunction and in research on novel approaches to the therapy of these pathologies. In particular, biologically active metabolites are being investigated that can produce compensating and therapeutic effects in various pathologies. One such compound is uridine, which plays an important role in the maintenance of cellular functions and energy metabolism. Uridine is a pyrimidine nucleoside composed of uracil and ribose. It is the most important component of RNA and glycogen synthesis. When interacting with phosphatidylcholine, uridine forms pyrimidine–lipid conjugates (phosphorus-containing compounds of choline or brain phospholipids) that participate in the synthesis of cell membranes [7]. Along with the participation in the synthesis of membranes, uridine derivatives, together with adenosine triphosphate and adenosine, operate in the nervous system as signaling molecules of processes such as neurogenesis, migration and differentiation of neurons, apoptosis, and proliferation of glial cells as well as being involved in synaptic transmission and neuromodulation [8]. Uridine and its derivatives have been widely used to diminish cytotoxicity, suppress drug-induced hepatic steatosis, and improve neurophysiological functions [9,10,11]. 

Uridine administration is known to prevent myocardial/brain damage in animal models of acute ischemia and ischemia/reperfusion through the restoration of the redox balance and the activation of the mitochondrial ATP-dependent channel [12,13,14].

Taking into account the beneficial effect of uridine on the cellular energy metabolism and redox balance, we assumed that nucleoside can serve as a therapeutic agent to correct metabolic disorders associated with HT. The goal of the present work is to estimate the therapeutic potential of uridine (intraperitoneal injection, 30 mg/kg for 7 days) and to study its effect on mitochondrial dysfunction in the liver of rats with induced HT.

## 2. Results

### 2.1. Effect of Uridine on Somatic and Biochemical Parameters of Rats with Hyperthyroidism

The development of HT was confirmed by measuring the concentration of free T_3_ and T_4_ in the serum of rats (Table 1). In experimental animals, the levels of T_3_ and T_4_ were elevated by 1.8 and 3.4 times, respectively, compared with the values in the control group. In addition, the injection of thyroxine to animals induced a decrease in the weight of the body and the liver (Table 1). The gain in bodyweight of HRs was reduced by more than twofold. 

The administration of uridine to HRs did not normalize the concentration of T_3_ and T_4_ in animals. At the same time, the weight and the body weight gain in Group HT + U returned to the norm compared with HRs (Table 1). The measurements of the activity of the main markers of liver damage, alanine aminotransferase (ALT), and aspartate aminotransferase (AST) showed an increase in the AST activity by 1.5 times in HRs. The injection of uridine did not affect the AST activity in the animals of Group HT + U. The activity of another enzyme used for the primary diagnosis of hepatic pathologies, lactate dehydrogenase (LDH), was also enhanced in HRs; in this case, uridine partially normalized this characteristic in the rats of Group HT + U (Table 1).

### 2.2. Effect of Uridine on the Functional State of Rat Liver Mitochondria with Hyperthyroidism

The next stage of the work consisted in studying the parameters of respiration and oxidative phosphorylation in liver mitochondria from the rats of the experimental groups (Table 2).

As it has been shown earlier, induced HT caused an increased oxygen consumption by liver mitochondria in different metabolic states; at the same time, the efficiency of ADP phosphorylation (ADP/O) and the respiratory control ratio (RCR) were decreased [15]. Table 2 also shows the respiration rates in different states (basal substrate respiration, ADP-stimulated respiration, and respiration at rest) in the examined rat liver mitochondria. In Groups HT and HT + U, these parameters were 25–70% higher compared with the same parameters in Groups C and C + U. Simultaneously, the RCR decreased by 1.3–1.4, indicating a reduction in the capacity of liver mitochondria for oxidative phosphorylation in Groups HT and HT + U. In this case, the administration of uridine together with T_4_ normalized ADP/O compared with this parameter in the rats of Group HT.

**Figure 1:**
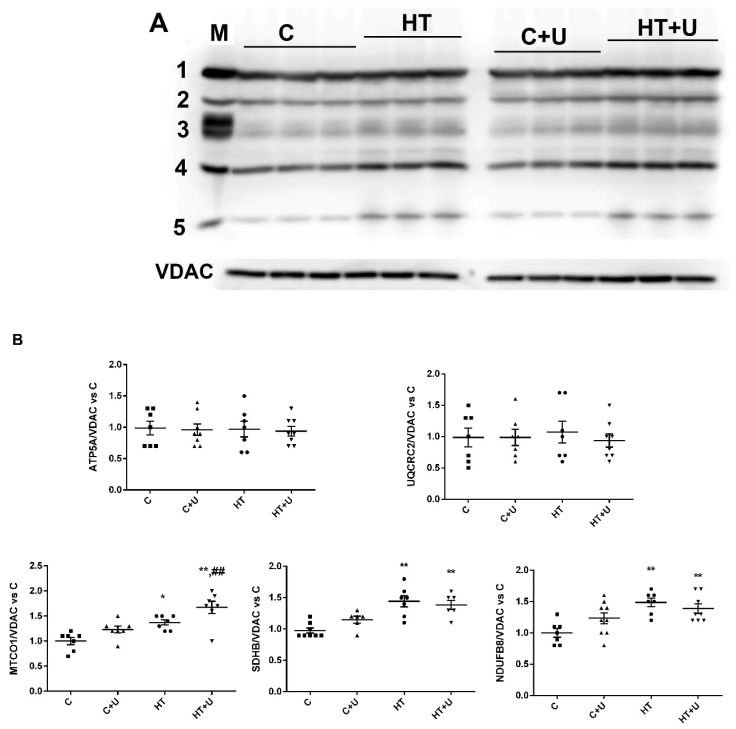
Level of the representative subunits of mitochondrial respiratory chain complexes. Data of the Western blot analysis (**A**) “M” indicates a positive control (rat liver mitochondrial lysate). Relative contents of Complex V (1, ATP5A)/VDAC ratio, Complex III (2, UQCRC2)/VDAC ratio, Complex IV (3, MTCO1)/VDAC ratio, Complex II (4, SDHB)/VDAC ratio, and Complex I (5, NDUFB8)/VDAC ratio (**B**) * *p* < 0.05 compared with Group C; ** *p* < 0.02 compared with Group C; and ^##^ *p* < 0.02 compared with Group C + U (*n* = 6–8 in each group).

To elucidate the reasons for changes in the mitochondrial respiration rates, we determined the content of proteins that form respiratory chain complexes in the mitochondria of the animals of the four experimental groups using the Western blot method. It is shown in Figure 1 that the level of the subunits of the CIV, CII, and CI complexes in the liver mitochondria from the rats of Groups HT and HT + U increased 1.3–1.6 times compared with the control animals. In the animals of Group HT + U, a significant increase in the level of the CIV subunit compared with this parameter in the rats of Group C + U was found. The administration of uridine to HRs (Group HT + U) did not change the level of respiratory chain complexes compared to the animals of Group HT. An insignificant increase in the content of OXPHOS proteins was noted in the liver mitochondria from the control rats, to which uridine was administered. The levels of CV and CIII of the mitochondrial respiratory chain in the animals of four experimental groups did not change (Figure 1).

The activation of respiration could also be due to an increased activity of the enzymes of the mitochondrial respiratory chain; therefore, we determined these parameters in the animals of the experimental groups (Table 3). It was found that the activity of complexes CI and CII as well as of citrate synthase (CS) in animals with HT increased 1.3 times compared with the control values. The activity of CS was also increased in the liver mitochondria of the rats of Group HT + U. On the other hand, the activity of CIII, CIV, and CV in HRs decreased 1.2–1.4 times compared with the control group. The injection of uridine to HRs led to an increase in the activity of almost all enzymes studied, except CIV, relative to those in the animals of Group C. Additionally, we observed an increase in the activities of CIII and CV by 1.6 and 1.3 times, respectively, compared with the animals in Group HT. After administering uridine to the rats of the control group, no significant differences in the activity of the enzymes of the ETC were found (Table 3).

**Figure 2:**
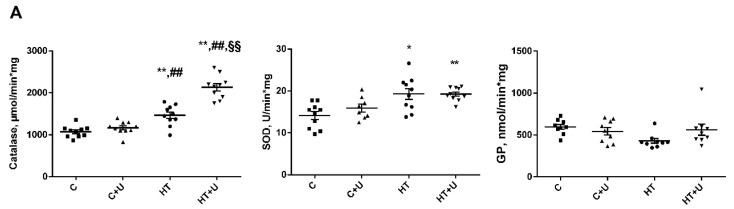

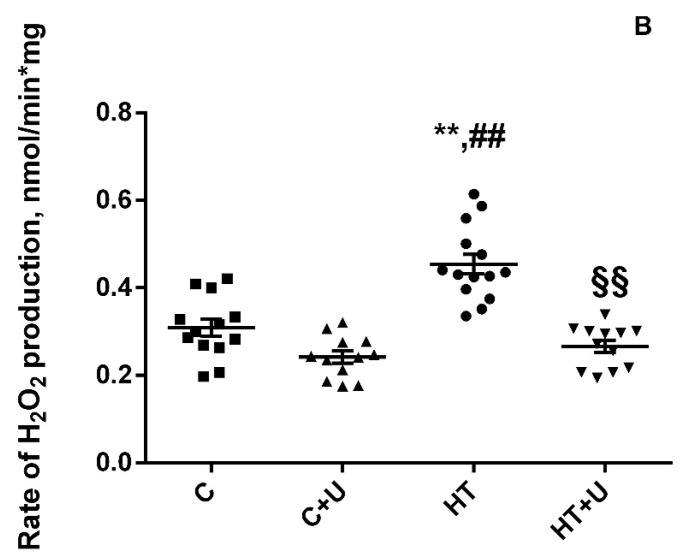
Activities of the antioxidant enzymes (**A**) and the rate of H_2_O_2_ production (**B**) in the liver mitochondria of the experimental animals. The substrate used consisted of 5 mM succinate + 5 mM glutamate. * *p* < 0.05 compared with Group C; ** *p* < 0.02 compared with Group C; ^##^ *p* < 0.02 compared with Group C + U; and ^§§^ *p* < 0.02 compared with Group HT (*n* ≥ 9 in each group).

It has been shown earlier that, in the liver mitochondria of HRs, oxidative stress develops [15]. In the present work, we examined how uridine affects antioxidative enzymes and the rate of H_2_O_2_ formation in rat liver mitochondria (Figure 2). It can be seen in Figure 2 that the activities of catalase (1460 ± 60 vs. 1088 ± 42 μmol/min·mg) and of total superoxide dismutase (SOD) increase (18.9 ± 1.4 vs. 14 ± 0.9 U/min·mg), and the activity of glutathione peroxidase (GP) tends to decrease compared with those of the control (597 ± 29 vs. 430 ± 28 nmol/min·mg). The administration of uridine to HRs further increased the activation of catalase compared to both the control and the group of animals with HT (2137 ± 54 vs. 1088 ± 42 and 1460 ± 60 μmol/min·mg). In addition, we observed a restoration of the activity of GP in the rats of Group HT + U to the value of the control group and, consequently, a tendency toward the correction of the activity of this enzyme compared with Group HT (562 ± 65 vs. 430 ± 28 nmol/min·mg). The activity of SOD after the injection of uridine in combination with thyroxine remained unchanged (19.3 ± 0.5 vs. 18.9 ± 1.4 U/min·mg). The administration of uridine to the animals of the control group did not affect the activity of the enzymes tested (Figure 2).

Oxidative stress always represents an imbalance between the formation and utilization of reactive oxygen species (ROS). Therefore, we measured the rate of H_2_O_2_ formation in the liver mitochondria of the animals of the experimental groups (Figure 2). The experimentally induced HT caused an increase in the rate of H_2_O_2_ formation of almost 1.5 times (0.48 ± 0.03 vs. 0.32 ± 0.02 nmol/min·mg). In the animals of Group HT + U, uridine decreased the rate of H_2_O_2_ production by 1.7 times compared to Group HT (0.27 ± 0.01 vs. 0.48 ± 0.03 nmol/min·mg, respectively). The administration of uridine to the animals of Group C + U led to a decrease in the H_2_O_2_ formation compared with the control animals (0.32 ± 0.02 vs. 0.24 ± 0.01 nmol/min·mg, respectively) (Figure 2).

### 2.3. Effect of Uridine on HT-Induced Changes in the Expression of mRNA of the Proteins Responsible for Biogenesis and Mitochondrial Quality Control

The decrease in the efficiency of ADP phosphorylation and the generation of oxidative stress in animals may be associated with changes in the biogenesis and dynamics of mitochondria that occur in the liver during the development of HT. In the subsequent stage of the study, we determined the level of the expression of genes that encode proteins involved in the fusion/fission of mitochondria, mitochondrial biogenesis, and mitophagy in the experimental groups. 

**Figure 3:**
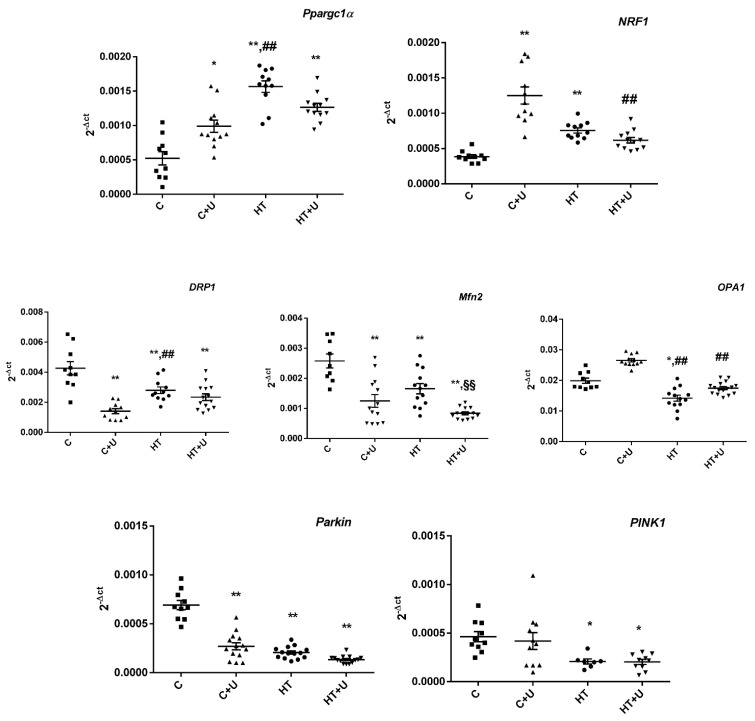
Analysis of mitochondrial homeostasis (fusion/fission, biogenesis, and mitophagy) in the liver of the experimental animals. The mRNA levels of the *Ppargc1a*, *NRF1*, *Drp1*, *Mfn2*, *OPA1*, *Pink1*, and *Parkin* genes in the liver of the rats in the experimental groups. * *p* < 0.05 compared with Group C; ** *p* < 0.02 compared with Group C; ^##^ *p* < 0.02 compared with Group C + U; and ^§§^ *p* < 0.02 compared with Group HT (*n* ≥ 10 in each group).

It can be seen in Figure 3 that HT was accompanied by an increase in the expression of *Ppargc1a* by almost three times compared to the value in the control samples. The level of the nuclear respiration factor 1 (*NRF1*) encoding the protein, which acts as a transcription factor and activates the expression of some key metabolic genes and nuclear genes necessary for the respiration, transcription, and replication of mitochondrial DNA, also increased, by almost twofold. Interestingly, the use of uridine in Group C + U induced an increase in the expression of both *Ppargc1a* and *NRF1* compared with the control, which may indicate an enhancement in the biogenesis of organelles. The administration of uridine to rats with HT did not change the expression of the genes *Ppargc1a* and *NRF1*. In this case, the mRNA level of *NRF1* in Group HT + U approached the value in the control but decreased compared with the value in Group C + U (Figure 3). The level of the expression of mRNA of the genes responsible for the dynamics of mitochondria in HRs was decreased compared to that of the control. The administration of uridine to rats with HT normalized the expression of the *OPA1* gene, but the expression of the genes *DRP1* and *Mfn2* decreased, as in Group HT. The level of *Mfn2* mRNA in the animals of Group HT + U significantly decreased compared to that of Group HT. In the rats of Group C + U, a decrease in the expression of the genes *DRP1* and *Mfn2*, and an insignificant increase in the expression of *OPA1* were observed. The evaluation of the expression of the genes responsible for mitophagy showed that the administration of uridine to HRs did not change the expression of the *Pink1* and *Parkin* genes compared to Group HT. The injection of uridine to the control rats led to an increase in the level of *Parkin* mRNA but did not affect the expression of *Pink1*.

## 3. Discussion

Considering the specific effect of TH on energy metabolism and taking into account the fact that mitochondria are the main cell components where metabolic events occur, it can be concluded that these organelles make a key contribution to the development of HT and hence become the central object of study. An excess of TH leads to the activation of a multitude of processes in the body, such as the considerable enhancement of biogenesis and changes in the content of the proteins responsible for the fusion and fission of mitochondria, the acute intensification of energy metabolism, and the development of oxidative stress [16]. The enhancement in the metabolism necessitates the identification and removal of damaged or nonviable organelles. In the process of HT, the impairment of mitophagy occurs, which causes the accumulation of damaged mitochondria; this in turn can promote cell death by releasing proapoptotic molecules and/or generating increased concentrations of ROS [4]. 

In some cases, the use of biologically active additives can reduce the severity of the disease and improve the quality of life of patients. One such nutraceutical is the nucleoside uridine, which plays an important role in the regulation of cell functions and energy metabolism, in particular, in the metabolism of carbohydrates, proteins, and lipids, as well as in the biosynthesis of nucleic acids [10,11,17].

In the present work, we assessed the effect of the long-term administration of uridine on a model of experimentally induced HT in rats. The determination of the content of T_3_ and T_4_, the primary diagnostic markers of the activity of the thyroid gland, showed a several-fold increase in their concentration in the blood serum, indicating the development of HT in animals (Table 1). The administration of uridine did not normalize the concentration of TH in the blood of HRs. Nevertheless, the dynamics of body weight in the hyperthyroid animals receiving uridine was stabilized compared with those of HRs. Presumably, this is related to the ability of uridine to affect the regulation of glycogen synthesis in the liver [18]. In addition, this nucleoside contributed to the maintenance of the integrity of liver cells, which is evidenced by a decrease in the activity of LDH in Group HT + U (Table 1).

TH stimulated an increase in oxygen consumption by the rat liver mitochondria with a simultaneous decrease in the metabolic activity (Table 2). The administration of uridine to the animals of Group HT + U did not affect the respiration rate but normalized the efficiency of oxidative phosphorylation compared to the animals with HT. This positive effect is probably associated with the possible participation of uridine in the synthesis of both acetyl-CoA and glycogen [19,20,21].

On the other hand, the hyperfunction of liver mitochondria in HRs could be caused by an increase in the level of OXPHOS enzymes, which is characteristic of the HT condition [13]. In the present study, as in the case described in [15], we recorded an increase in the levels of complexes CIV, CII, and CI in the rats of Group HT. In this case, the combined administration of T_4_ and uridine did not change the content of the proteins of the ETC, and the levels of CIV, CII, and CI remained elevated (Figure 1). Nevertheless, the administration of uridine to the animals of Group HT led to the correction of the activity of some complexes, namely, the restoration of the normal operation of CV and a tendency toward the normalization of the activity of complexes CII+III and CIV (Table 3). It is also noteworthy the increase in the activity of complex CIII in Group HT + U compared to this parameter in the other groups of animals. Taking into consideration all the data on energy metabolism, it can be said that uridine partially prevented the defects caused by HT.

The mitochondrial respiratory chain is the main intracellular source of ROS. In the HT model used, the efficiency of electron transfer could be impaired due to the inhibition of the complexes CIII and CIV. In addition, the elevated activity of CI and CII could greatly increase the level of superoxide radical and facilitate the development of oxidative stress in the whole. It is known that some beneficial effects of uridine in ischemic heart damage, as well as in other cellular pathologies associated with oxidative stress, can be due to the action of the nucleoside on the system of cell defense against ROS [22,23]. Therefore, we assessed the effect of uridine on the development of oxidative stress in rat liver mitochondria with the HT condition. As shown in Figure 2, the activity of SOD was increased, which indirectly indicates a greatly enhanced formation of the substrate of this enzyme, superoxide radical, both in the HT and HT + U groups. At the same time, the activity of GP in the liver of HRs tended to decrease, whereas the treatment with uridine normalized the activity of the enzyme to control values (Figure 2). Although catalase has a low affinity to H_2_O_2_ compared with GP [24], its activity in Group HT + U significantly increased compared with the other experimental groups, which indicates a compensatory hyperactivity of this enzyme in inhibiting the development of oxidative stress in HT. As to the rate of H_2_O_2_ production, the administration of uridine decreased it in the liver mitochondria of HRs (Figure 2). Thus, these data confirm, once more, the antioxidant action of this nucleoside in various diseases. 

The restoration of the normal values of the parameters of oxidative phosphorylation, such as the RCR and ADP/O, in the liver mitochondria of the animals after the administration of uridine may be related to the normalization of the functioning of the systems responsible for mitochondrial biogenesis and mitochondrial quality control. It is known that HT causes the activation of mitochondrial biogenesis, changes in the content of the proteins involved in mitochondrial dynamics, and the inhibition of PINK1/Parkin-dependent mitophagy in the heart and liver of rats [4,6,25,26]. We found an increase in the expression of the *Ppargc1a* and *NRF1* genes in the animals of Group C + U, which may indicate that uridine can affect the mitochondrial biogenesis and the expression of a great number of genes encoding proteins that regulate oxidation reactions and the carbohydrate/fat metabolism, not only in pathological but also in normal conditions (Figure 3). The injection of the nucleoside in combination with T_4_ did not affect the increased mRNA levels of *Ppargc1a* and *NRF1*. In this case, the level of expression of the *NRF1* gene approached the control value but was significantly lower than the value in Group C + U. As for the expression of the *DRP1* gene responsible for mitochondrial fission, uridine lowered this parameter in the animals of the C + U and HT + U groups, which may indirectly suggest the inhibition of the organelle fission process (Figure 3). In turn, a decrease in the *Mfn2* mRNA level in these same groups may, probably, indicate a disturbance in the process of the fusion of the outer mitochondrial membrane. In this case, the expression of the *Mfn2* gene in the animals of Group HT + U was even lower than that in Group HT. The administration of uridine to the animals of Group C diminished the expression of the *Mfn2* and *DRP1* genes but did not affect the mRNA level of *OPA1*. The expression of the *OPA1* gene was reduced in the HT condition; however, after exposure to uridine, this parameter was normalized to the control value, which indirectly indicates that nucleoside maintains the structural organization and integrity of the inner mitochondrial membrane to optimize the function of mitochondria with increased demands for energy in HT (Figure 3). As shown in Figure 3, the combined administration of uridine with T_4_ did not affect the reduced expression of the *Pink1* and *Parkin* genes responsible for the process of mitophagy. In this case, uridine did not positively affect the removal of damaged mitochondria in the HT condition. Thus, the use of uridine can enhance mitochondrial biogenesis in the animals of Group C but partially weaken the process of fusion and division of organelles and can also have no effect on the biogenesis and mitophagy in the liver of hyperthyroid rats.

## 4. Materials and Methods

### 4.1. Experimental Animals

Male rats of the Wistar line weighing 210–240 g were used. Animals were arbitrarily divided into four groups: (1) saline-treated control (C); (2) control + uridine (C + U); (3) animals to which a solution of T_4_ was administered (HT); and (4) rats to which a solution of T_4_ and 15 min later uridine were injected (HT + U). Hyperthyroidism (HT) was modeled by the intraperitoneal injection of T_4_ (Sigma-Aldrich, St. Louis, MO, USA), as the most stable hormone in solution, for 7 days at a concentration of 200 µg per 100 g body weight. The rats in Groups C + U and HT + U were treated with uridine at a dose of 30 mg/kg by intraperitoneal injection for 7 days. Uridine (Sigma-Aldrich, St. Louis, MO, USA) was dissolved in sterile saline immediately prior to injections. At the end of the treatment, all rats were sacrificed, and body/liver weights were recorded. The levels of free T_3_ and T_4_, lactate dehydrogenase, alanine aminotransferase, and aspartate aminotransferase were determined by serum using the appropriate reagent kits (Vector-Best, Novosibirsk, Russia).

### 4.2. Isolation of the Liver Mitochondria and Assessment of Mitochondrial Functions in the Rats of the Experimental Groups

Mitochondria were isolated from the liver by differential centrifugation as described earlier [15]. The isolation medium contained 210 mM mannitol, 70 mM sucrose, 10 mM HEPES-KOH, 0.1% fatty acid-free BSA, and 0.5 mM EGTA (pH 7.4). The concentration of the protein was determined by the Lowry method [27]. 

The rate of O_2_ consumption by isolated mitochondria was estimated by high-resolution respirometry with an Oroboros Oxygraph-2k device (Oroboros Instruments, Innsbruck, Austria). The reaction medium contained 100 mM sucrose, 50 mM mannitol, 60 mM KCl, 5 mM potassium glutamate, 5 mM potassium malate, 10 mM HEPES, 0.5 mM EGTA-K, and 2.5 mM KH_2_PO_4_ (pH 7.4). Other reagents included 0.2 mM ADP and 50 µM 2,4-dinitrophenol (DNP). The rates of substrate oxidation (V_2_, the rate in state 2; V_3_, the rate in state 3; V_4_, the rate in state 4; and V_DNP_, the rate in uncoupled respiration state) were expressed as ng-atoms O/min·mg of mitochondrial protein. Respiratory control ratios (RCR = state_3_/state_4_), respiratory states, and ADP/O ratios were determined according to Chance and Williams [28].

The rate of H_2_O_2_ production was determined by the fluorescent dye Amplex red (excitation wavelength 560 nm; emission wavelength 590 nm) using a Tecan Spark 10 M plate reader (Tecan, Männedorf, Switzerland) at 37 °C under constant stirring. The incubation medium contained 100 mM sucrose, 50 mM mannitol, 65 mM KCl, 10 mM HEPES, 0.5 mM EGTA-K, and 2.5 mM KH_2_PO_4_ (pH 7.4); respiration substrates included 2 U/mL of peroxidase, 10 µM Amplex red, and 0.2 mg/mL of the mitochondrial protein. The amount of the resulting hydrogen peroxide was calculated from the calibration curve. A standard hydrogen peroxide solution was prepared on the day of experiment; its concentration was determined using the molar absorption coefficient E_240_ = 43.6 M^−1^ cm^−1^ [5].

#### 4.2.1. Enzyme Activity Assay

The enzyme activity was determined in mitochondria osmotically disrupted in 5 mM potassium phosphate buffer (pH 7.4) at 4 °C for 15 min and subjected to three freezing and thawing cycles. The activity of soluble enzymes was measured in the supernatant. The precipitate was lysed in 0.5 mL of a lysis mixture (5 mM potassium phosphate buffer, pH 7.4; protease inhibitor cocktail), and membrane-linked enzyme activity was determined in the lysate. 

The activities of the respiratory chain complexes were determined in mitochondrial preparations as described in [29] with modifications [15].

#### 4.2.2. SOD Activity

SOD activity was measured spectrophotometrically at 550 nm and 25 °C as the rate of inhibition of nitroblue tetrazolium reduction in a xanthine–xanthine oxidase system [30]. The activity unit (U) of SOD was taken as the enzyme quantity inhibiting the reduction in nitroblue tetrazolium by 50%. Specific activity was defined as U/mg protein.

#### 4.2.3. CAT Activity

The catalase activity was estimated by measuring changes in absorbance at 240 nm and 25 °C using H_2_O_2_ as a substrate and expressed as nmol/min·mg [31].

#### 4.2.4. GPX Activity

The activity of GP was estimated by measuring the decrease in absorbance at 340 nm due to the NADPH oxidation in the presence of H_2_O_2_ and GSH and expressed as nmol/min·mg protein [32].

### 4.3. Quantification of the Expression of the Genes of Mitochondrial Dynamics, Biogenesis, and Mitophagy Using Quantitative Real-Time PCR in the Rats of the Experimental Groups

The total RNA was isolated from 100 mg of deep-frozen tissue samples from the liver using an ExtractRNA kit (#BC032, Eurogen, Moscow, Russia) in accordance with the manufacturer’s protocols. The total RNA concentration was measured spectrophotometrically using an Implen NanoPhotometer C40 instrument (Implen GMBH, München, Germany). The real-time PCR was performed on a QuantStudio 1 system (Thermo Fisher Scientific, Waltham, MA, USA) using the qPCRmix-HS SYBR reaction mixture (Eurogen, Moscow, Russia). The selection and analysis of gene-specific primers were carried out using the Primer-BLAST software (https://www.ncbi.nlm.nih.gov/tools/primer-blast/) [33] (the oligonucleotide sequences are presented in Table 4). The differences between the experimental and control values were calculated according to the formula ∆Ct = Ct (tested gene) − Ct (*Actb*) [34].

### 4.4. Isolation of Rat Liver Mitochondria and Determination of Respiration and Oxidative Phosphorylation Electrophoresis and Immunoblotting of Mitochondrial OXPHOS Proteins

Samples for electrophoresis were prepared from rat liver mitochondria stored at −80 °C using a Complete Protease Inhibitor Cocktail (P8340, Sigma-Aldrich, St. Louis, MO, USA), Phosphatase Inhibitor Cocktail II (ab201113, Abcam, Cambridge, UK), PMSF (1 mM), EGTA (1 mM), and EDTA (1 mM). Proteins at equal concentrations were applied to each lane and subjected to electrophoresis followed by Western blot analysis. The mitochondrial samples were separated by 10% SDS-PAGE and transferred to a 0.45 µm nitrocellulose membrane (Cytiva, Marlborough, MA, USA). After overnight blocking, the membrane was incubated with the appropriate primary antibody. The immunoreactivity was determined using the appropriate secondary antibody conjugated to horseradish peroxidase (7074, Cell Signaling Technology Inc., Danvers, MA, USA). The Rat Heart Mitochondria Control (ab 110413, Waltham, MA, USA) was used as a Western blot control. Peroxidase activity was estimated with ECL chemiluminescence reagents (Pierce, Rockford, IL, USA). The relative levels of the detected proteins were visualized using an LI-COR system (LI-COR, Lincoln, NE, USA) and normalized to the VDAC loading control. Optical density measurements were performed by the LI-COR Image Studio software 4.0.21. The antibodies used were (ab110413) the total OXPHOS Rodent WB Antibody Cocktail and (ab154856) VDAC antibody.

### 4.5. Statistical Analysis

The data were analyzed using the program GraphPad Prism 6.0 (GraphPad Software Inc., San Diego, CA, USA) and Excel software 15.0.5357 and are presented as means ± SEM of 6–15 independent experiments in each group. The normality of the distribution of variables was tested using the Shapiro–Wilk test. A one-way ANOVA (Tukey’s multiple comparison post hoc test) was performed for the normally distributed data. For non-normally distributed data, a one-way ANOVA using the Kruskal–Wallis test was used.

## 5. Conclusions

In the present work, the effect of chronic exposure to uridine (30 mg/kg for 7 days) on the development and concomitant mitochondrial dysfunction in the liver of rats with experimentally induced HT (200 µg T_4_/100 g body weight for 7 days) was shown. Based on the data obtained, the following conclusions can be made: (1) uridine restores the body weight in the HT condition; (2) uridine improves the capacity of mitochondria for oxidative phosphorylation but has no significant effect on the level of the proteins of the mitochondrial system OXPHOS in HR; (3) uridine normalizes the activity of the complex CV; (4) the administration of uridine prevents the development of oxidative stress in liver mitochondria from hyperthyroid animals; and (5) uridine affects mitochondrial biogenesis by increasing the expression of the *Ppargc1a* and *NRF1* genes and decreasing the expression of the *Parkin* gene responsible for the process of mitophagy in the control animals. The injection of uridine to HRs had no effect on the mRNA level of the *Ppargc1a*, *NRF1*, *Parkin,* and *PINK1* genes. In addition, the level of *OPA1* mRNA was restored, indicating a normalization of the activity of the ETC and oxidative phosphorylation in the mitochondria of hyperthyroid rats. The dose of uridine and the duration of uridine injection used in the work had no side effects and no adverse effects on the animals. The data obtained permit one to characterize uridine as a natural metabolite capable of reducing the severity of systemic consequences of the disease and partially preventing bioenergetic disorders and oxidative stress in the liver of rats with experimentally induced HT. The question of how uridine affects the mitochondrial dysfunction needs further research, but even now it can be said that this nucleoside can be considered as part of the therapy to improve the quality of life in this pathology.

## Figures and Tables

**Table 1:** Somatic and biochemical parameters in the animals of the experimental groups.

	C	C + U	HT	HT + U
T_3 free_, pmol/L	5.2 ± 0.3	5.8 ± 0.4	9.3 ± 1.2 **	10 ± 1 **^, ##^
T_4 free_, pmol/L	19.2 ± 1.0	19.1 ± 2.9	66.2 ± 4.4 **	70 ± 8 **^, ##^
Body weight, g	272 ± 3.8	292 ± 8.3	238 ± 5.7 **^, ##^	271 ± 6.8 ^§^
Liver weight, g	12.4 ± 0.4	12.1 ± 0.6	8.7 ± 0.4 **^, ##^	9.8 ± 0.6 *^, #^
Body weight gain, g	42.6 ± 5.1	56 ± 4.5	14.3 ± 1.4 **^, ##^	30 ± 4.2 ^§, ##^
ALT, µmol/min·mg	42.7 ± 2.1	35 ± 1.1	43.9 ± 3.6	47 ± 2.4
AST, µmol/min·mg	65.5 ± 1.7	75.4 ± 1.5	106.7 ± 14 **^, ##^	105.4 ± 6 **^, #^
LDH, µmol/min·mg	258 ± 6.4	292 ± 16	393 ± 30 **^, ##^	316 ± 11 ^§^

* *p* < 0.05 compared with Group C; ^#^ *p* < 0.05 compared with Group C + U; ** *p* < 0.02 compared with Group C + U C; ^##^ *p* < 0.02 compared with Group C + U C + U; ^§^ *p* < 0.05 compared with Group C + U HT (*n* ≥ 10–25 in each group).

**Table 2:** Parameters of respiration and oxidative phosphorylation of the rat liver mitochondria in the experimental groups.

	State _2_	State _3_	State _4_	State _DNP_	RCR	ADP/O
C	3.7 ± 0.3	41.3 ± 0.8	4.5 ± 0.2	41 ± 0.8	9.2 ± 0.2	2.9 ± 0.04
C + U	3.4 ± 0.2	41.5 ± 0.8	4.6 ± 0.2	43 ± 1.3	9.1 ± 0.2	2.9 ± 0.02
HT	5.9 ± 0.4 **^, ##^	52 ± 1.3 **^, ##^	7.9 ± 0.4 **^, ##^	47 ± 1.8	6.6 ± 0.3 **^, ##^	2.6 ± 0.04 **^, ##^
HT + U	5.6 ± 0.3 **^, ##^	51.4 ± 1.3 **^, ##^	7.2 ± 0.3 **^, ##^	47 ± 1.6	7.2 ± 0.2 **^, ##^	2.9 ± 0.03 ^§§^

States _2_, _3_, _4_, _DNP_—nmol O_2_/min·mg of protein; ADP/O—µmol/ng atoms O. Additions: 5 mM glutamate + 5 mM malate, 0.2 mM ADP, 0.05 mM DNP, Mitochondrial protein: 1 mg/mL. ** *p* < 0.02 compared with Group C; ^##^ *p* < 0.02 compared with Group C + U; and ^§§^ *p* < 0.02 compared with Group HT (*n* = 15–20 in each group).

**Table 3:** Activities of the enzymes of the ETC and citrate synthase in the liver mitochondria from the experimental groups.

	C	C + U	HT	HT + U
CS, nmol/min·mg	222 ± 11	250 ± 6	300 ± 9 **^, ##^	299 ± 9 **^, ##^
CI, nmol/min·mg	101 ± 8	125 ± 8	135 ± 7 *	145 ± 11 **
CII, nmol/min·mg	24 ± 0.6	29 ± 2.7	32 ± 1 **	36 ± 3.4 **
CII+III, nmol/min·mg	240 ± 18	200 ± 20	185 ± 13	233 ± 27
CIII, nmol/min·mg	400 ± 19	470 ± 19	320 ± 20 *^, ##^	506 ± 54 *^, §§^
CIV, nmol/min·mg	1453 ± 91	1265 ± 85	1028 ± 117 **	1173 ± 70
CV, nmol/min·mg	820 ± 25	899 ± 53	701 ± 29 *^, #^	922 ± 96 ^§§^

* *p* < 0.05 compared with Group C; ^#^ *p* < 0.05 compared with Group C + U; ** *p* < 0.02 compared with Group C; ^##^ *p* < 0.02 compared with Group C + U; and ^§§^ *p* < 0.02 compared with Group HT (*n* ≥ 10 in each group).

**Table 4:** List of gene-specific primers for the real-time PCR analysis.

Gene	Forward (5′ → 3′)	Reverse (5′ → 3′)
*Drp1*	GATCCAGATGGGCGCAGAAC	ATGTCCAGTTGGCTCCTGTT
*Mfn2*	AGCGTCCTCTCCCTCTGACA	TTCCACACCACTCCTCCGAC
*OPA1*	GCAGAAGACAGCTTGAGGGT	TGCGTCCCACTGTTGCTTAT
*PINK1*	GATGTGGAATATCTCGGCAGGA	TGTTTGCTGAACCCAAGGCT
*Parkin*	GGCCAGAGGAAAGTCACCTG	CACCCGGTATGCCTGAGAAG
*Ppargc1α*	TGACATAGAGTGTGCTGCCC	GCTGTCTGTGTCCAGGTCAT
*Nrf1*	TACAAGGCGGGGGACAGATA	TGCATGAACTCCATCTGGGC
*Actb*	GACCCAGATCATGTTTGAGACCT	CCAGAGGCATACAGGGACAAC

## Data Availability

The data presented in this study are available upon request from the corresponding authors.
